# Profiling of CSF: Reliability of Diagnosis

**DOI:** 10.1371/journal.pmed.0030469

**Published:** 2006-10-31

**Authors:** Robert Matthews

Research that points towards more reliable diagnosis of schizophrenia is always to be welcomed. However, the impact of the relatively low prevalence of this disorder on the reliability of any test should be borne in mind when assessing any proposed diagnostic.

Holmes et al. [1] find a sensitivity for their cerebrospinal fluid (CSF) test of 82% and a specificity of 85%. Thus their test increases the weight of evidence in favour of a diagnosis of schizophrenia by a factor of 0.82/(1 - 0.85) = 5.5. While impressive, this figure must be balanced against the population prevalence of schizophrenia of around 1%. A simple calculation then shows that the CSF test increases the probability for the presence of schizophrenia to around 5%. Or, put somewhat more bleakly, in the absence of any other diagnostic evidence, it is still 95% probable that a diagnosis of schizophrenia is not merited. While the work of Holmes et al. may well be a step towards reliable diagnosis, it is perhaps a much smaller step than one might expect.
